# Analysis of the Impact of the Confinement Resulting from COVID-19 on the Lifestyle and Psychological Wellbeing of Spanish Pregnant Women: An Internet-Based Cross-Sectional Survey

**DOI:** 10.3390/ijerph17165933

**Published:** 2020-08-15

**Authors:** Gemma Biviá-Roig, Valentina Lucia La Rosa, María Gómez-Tébar, Lola Serrano-Raya, Juan José Amer-Cuenca, Salvatore Caruso, Elena Commodari, Antonio Barrasa-Shaw, Juan Francisco Lisón

**Affiliations:** 1 Departamento de Fisioterapia, Facultad de Ciencias de la Salud, Universidad Cardenal Herrera-CEU, CEU Universities, C/Ramón y Cajal, 20, Alfara del Patriarca, 46115 Valencia, Spain; gemma.bivia@uchceu.es (G.B.-R.); juanjoamer@uchceu.es (J.J.A.-C.); 2 Department of Educational Sciences, University of Catania, 95124 Catania, Italy; valarosa@unict.it (V.L.L.R.); e.commodari@unict.it (E.C.); 3 Obstetrics and Gynecology Service, Hospital of Manises, 46940 Valencia, Spain; mllanos@hospitalmanises.es; 4 Obstetrics and Gynecology Service, Hospital of Sagunto, 46500 Valencia, Spain; serrano_marray@gva.es; 5 Obstetrics and Gynecology Unit, Department of General Surgery and Medical Surgical Specialties, University of Catania, 95123 Catania, Italy; scaruso@unict.it; 6 Departamento de Cirugía, Facultad de Ciencias de la Salud, Universidad Cardenal Herrera-CEU, CEU Universities, C/Ramón y Cajal, 20, Alfara del Patriarca, 46115 Valencia, Spain; 7 Departamento de Medicina, Facultad de Ciencias de la Salud, Universidad Cardenal Herrera-CEU, CEU Universities, C/Ramón y Cajal, 20, Alfara del Patriarca, 46115 Valencia, Spain; juanfran@uchceu.es; 8 CIBER of Physiopathology of Obesity and Nutrition CIBERobn, CB06/03 Carlos III Health Institute, 28029 Madrid, Spain

**Keywords:** pregnancy, COVID-19, quarantine, lifestyle, quality of life

## Abstract

(1) Background: This study aimed to analyze the impact of the confinement due to the COVID-19 pandemics on the eating, exercise, and quality-of-life habits of pregnant women. (2) Methods: This was an internet-based cross-sectional survey which collected information about adherence to the Mediterranean diet, physical exercise, health-related quality of life (HRQoL), and perceived obstacles (in terms of exercise, preparation for delivery, and medical appointments) of pregnant women before and after the confinement. The survey was conducted in 18–31 May 2020. (3) Results: A total of 90 pregnant women participated in this study. There was a significant decrease in the levels of physical activity (*p* < 0.01) as well as in HRQoL (*p* < 0.005). The number of hours spent sitting increased by 50% (*p* < 0.001), 52.2% were unable to attend delivery preparation sessions because these had been cancelled. However, there were no significant differences in the eating pattern of these women (*p* = 0.672). *Conclusions*: These results suggest the need to implement specific online programs to promote exercise and reduce stress, thus improving the HRQoL in this population, should similar confinements need to occur again for any reason in the future.

## 1. Introduction

After the first outbreak of the disease caused by the SARS-CoV-2 virus in Wuhan (China) in December 2019, its rapid spread led the WHO to declare a global health emergency, and shortly after that, it came to be considered a pandemic [1]. The state of alarm was declared in Spain on 14 March 2020, with 5753 reported cases, resulting in the confinement of every citizen during this period [2]. The strict measures of social distancing and isolation at home used to contain the disease’s spread meant that the usual routines of the population, including every pregnant woman, were drastically modified. 

The lifestyle of pregnant women has a fundamental impact on maternal health and fetal development [3,4]. During pregnancy, the development of maternal tissues, fetal growth, and milk production increase women’s nutritional requirements [5,6,7,8]. Thus, pregnant women are encouraged to adhere to healthy dietary patterns, such as the Mediterranean diet (MD), which is currently considered one of the world’s healthiest diets [9,10]. This diet is characterized by the consumption of high quantities of vegetables, legumes, fruits, and nuts, unrefined cereals, fish, and olive oil, a low to moderate intake of dairy products, and consumption of low amounts of red meat, poultry, and saturated fats [11]. However, the consumption of inadequate diets with excessive amounts of foods with added sugar and/or a deficit of certain macronutrients and vitamins have also been described in developed countries, such as Spain and Italy [12]. 

On the one hand, an inadequate diet during pregnancy can predispose women to become overweight or obese and encourage the development of various complications and maternal-fetal pathologies. These include gestational diabetes, preeclampsia, instrumented vaginal deliveries, cesarean section, fetal macrosomia, or premature births [13,14,15,16,17,18,19,20,21]. Stressful situations, such as those produced by this new confinement situation, can be the cause of overeating and uncontrolled anxiety towards food, especially foods rich in sugar [22,23]. Indeed, some studies suggest that psychological and emotional responses to the COVID-19 outbreak [24,25] may have also increased the risk of developing dysfunctional eating behaviors and overeating as a consequence of boredom [26,27]. In this sense, the consumption of foods rich in sugar can reduce stress by stimulating the production of serotonin, the "feel-good” hormone [28]. However, uncontrolled food consumption is causally related to the development of cardiovascular disease and obesity.

On the other hand, engaging in regular physical activity and exercise during pregnancy is positively related to an increase in the general state of health of both the mother and the fetus [29,30,31]. These benefits include a lower risk of excessive gestational weight gain [32], gestational diabetes, preterm delivery, varicose veins, venous thrombosis, dyspnea [33,34,35,36], and postpartum depression [37]. On the contrary, there is also scientific evidence that supports the negative impact of physical inactivity and a sedentary lifestyle on general health [38], and in particular on the health of pregnant women [39,40]. For this reason, the clinical guidelines recommend that healthy women should engage in moderate physical activity during pregnancy, which combines aerobic exercise and muscular strength work for 150 min per week, or at least 20 to 30 min daily [41]. However, some researchers have observed that many pregnant women (between 32% and 96%) do not achieve these recommended levels and that these levels decrease as the pregnancy progresses [42]. 

Given the above, pregnant women could find a situation of confinement alarming, especially considering that the vast majority of them choose to walk as their primary physical activity [43,44]. Considering the significant mobility restrictions imposed by the Spanish government regarding outdoor activities during the recent confinement, we would expect there to have been a considerable reduction in the levels of physical activity in this population group with consequent repercussions on their state of health, as described above. Furthermore, in this new context, many pregnant women showed high levels of stress and concern during the COVID-19 pandemic, with one of the main reasons for this fear being that of possible contagion in medical and hospital centers [45]. This fact, together with a drastic change in these women’s usual routines, could result in a significant decrease in their health-related quality of life (HRQoL). Given the importance of lifestyle on health, the WHO published a guide with several recommendations for coping with home confinement. It highlighted the importance of maintaining healthy eating habits and staying physically active by following an exercise routine [46]. Thus, in light of these considerations, the main objective of this study was to collect and analyze data related to the impact of confinement on adherence of pregnant women to the MD, physical exercise engagement, and HRQoL during the COVID-19 pandemic. 

## 2. Materials and Methods

We conducted an internet-based cross-sectional survey. The survey was disseminated among pregnant women through midwives and gynecologists attending patients in Valencia (Spain). The participants were provided with an internet link to the survey created with the Google Forms application (Google LLC, Mountain View, CA, USA). This tool allows the creation of online surveys and collects the data automatically in a spreadsheet. The completion of online questionnaires is an established method for use in healthcare research [47]. It was chosen for its practicality and the simplicity of its distribution method during this particular situation of confinement that imposed significant restrictions on individual mobility. The data were collected from 18 May to 31 May (the last fortnight of strict confinement in Spain) after two months of prior confinement. The participants filled out the survey only once. They were asked to answer questions about their habits both before and during the confinement to analyze possible changes in the study variables.

### 2.1. Recruitment

The inclusion criteria were pregnant women aged over 18 years who were in the second or third trimester of their pregnancy. Women with recent pregnancies in the first trimester were excluded because it would have been impossible to obtain data related to these cases during pregnancy from before confinement.

The online questionnaire comprised 36 items and collected information about the following:

- Anthropometric (age, height, and weight) and obstetric (week of gestation) data.
- Perceived obstacles to physical exercise and care in preparing for childbirth.
- Eating habits using the Mediterranean Diet Adherence Screener (MEDAS) questionnaire from the PREDIMED study. This questionnaire assesses adherence to the MD and has been validated for the Spanish population. The MEDAS comprises 14 items, 12 of them on the frequency of food consumption and 2 on the dietary habits characteristics of the Spanish MD; each item is assigned a value of 0 or 1. Based on the score obtained, the participants were classified as ‘low adherence’ (score between 0–5), ‘medium adherence’ (score between 6–9), or ‘high adherence’ (score ≥ 10) [48].
- Physical activity level: general information was collected on the level of physical activity these women were engaging in (according to their perception of the exercise they had performed), as well as specific information, such as the number of days per week and minutes per day they had performed vigorous or moderate exercise or had walked. Following the WHO’s guidelines [49], vigorous exercise was considered to require much effort and to cause rapid breathing and a substantial increase in heart rate, such as lifting heavy weights, climbing stairs, swimming, or fast pedaling, running, sports such as soccer, individual tennis, HIIT or high-intensity exercise circuits, Zumba, and similar activities. Moderate exercise was considered to be that which perceptibly accelerated the heart rate, such as lifting light weights, swimming, pedaling at a regular speed, dance, or housework such as sweeping.
- Their level of sedentary behavior, which included the number of hours they sat per day, including the time spent sitting at a desk, reading, or sitting or lying down watching television.
- HRQoL was assessed using the three-level EuroQol-5D questionnaire (EQ-5D-3L). This tool has been widely used worldwide in various studies [50] and has been validated for use in the Spanish population [51]. The questionnaire has two parts: (1) The EQ-5D-3L tool. This is a descriptive system that includes five different health dimensions: mobility, self-care, usual activities, pain or discomfort, and anxiety or depression. Each dimension has three levels of severity: level 1 (no problems), level 2 (some problems), and level 3 (extreme problems). The participant was asked to indicate her state by checking the box aligned with the most appropriate statement in each of five dimensions. (2) The EQ-VAS (primary outcome) is a vertical analogue visual scale that ranges from 0 (worst imaginable state of health) to 100 (best imaginable state of health). The participant was asked to mark the vertical line’s point that best reflected her assessment of her health.

### 2.2. Ethical Considerations

This study was approved by the Ethics Committee at the CEU Cardenal-Herrera University (CEI20/069) and followed the fundamental principles established in the Declaration of Helsinki. All the participants were informed about the characteristics of the study before agreeing to participate. The anonymous response to the survey was completely voluntarily. The participants had to check a specific box to give their consent to participate in the study.

### 2.3. Statistical Analysis

There are no prospective data on quality-of-life habits of pregnant women during confinement. The following assumptions were made to test the hypothesis that the quality-of-life habits is lower during confinement: the EQ-VAS levels are significantly decreased during confinement. We performed a power analysis using the G*Power (v3.1.9.2, Heinrich-Heine-Universität Düsseldorf, Germany) program and found that 90 participants would provide 80% statistical power at a 5% significance level (two-sided test) for a small to medium effect size (d = 0.3).

After testing the normality of the data using the Shapiro–Wilk test, the nonparametric Wilcoxon tests were carried out to compare the pre- and during COVID-19 confinement values of the level of physical activity (vigorous, moderate, walking activities, and the number of hours sitting per day) and the EQ-VAS. In addition, Chi-square tests were carried out to assess the association between categorical variables (adherence to the Mediterranean Diet, mobility, self-care, usual activities, pain or discomfort, and anxiety or depression). The data are represented as a number and percentage shown in parentheses for categorical variables, or medians and interquartile ranges in square brackets for continuous variables. The data analysis was performed with SPSS software (version 22.0, SPSS Inc., Chicago, IL, USA) for Windows (IBM Corp., Armonk, NY, USA). We used a significance level of *p* < 0.05 for all of the statistical tests.

## 3. Results

The web-survey was concluded on 31 May 2020; the questionnaire was sent to 133 pregnant women and a total of 97 participants (72.9%) completed it, and after validation of the data, 90 respondents were finally included in the study (seven questionnaires were excluded because the participants were in the first trimester of their pregnancy).

The general characteristics (mean ± standard deviation) of the participants are shown in Table 1.

Table 2 shows their self-perception and perceived obstacles to exercise, preparation for delivery, and medical monitoring of their pregnancy during the confinement.

Our results showed significant decreases in physical activity (vigorous, moderate, and walking activities) and EQ-VAS scores, and significant increases in the number of hours the participants sitting per day during COVID-19 confinement compared to the pre-COVID-19 period (Table 3). Indeed, the results from the EQ-VAS test showed a large effect size (d = 0.82).

Regarding the EQ-5D, the results showed a significant decline in the five dimensions during the confinement compared to the pre-COVID-19 period (mobility, *p* < 0.001; self-care, *p* = 0.003; usual activities, *p* < 0.001; pain or discomfort, *p* < 0.001; and anxiety or depression, *p* < 0.001; Table 4). In terms of adherence to the MD (low, medium, or high), there were no significant differences between the pre- and during COVID-19 confinement values (*p* = 0.672; Table 5).

Table 5 also shows the answers to the 14-items on the MEDAS questionnaire.

A daily serving of vegetables is: 1 medium portion = 200 g. Fruit daily serving: 1 serving = 100–150 g portion. Red meat/hamburgers/other meat daily serving: 1 medium portion = 100–150 g. Butter, margarine, or cream daily serving: 1 medium portion = 12 g. Sweet or sugar-sweetened carbonated beverages daily serving: 1 medium portion = 200 mL. Wine daily serving: 1 medium portion = 125 mL. Legumes weekly serving: 1 portion = 150 g. Fish daily serving: 1 medium portion = 100–150 g. Seafood daily serving: 1 medium portion = 200 g. Nuts weekly serving: 1 portion of dairy products daily serving = 30 g

## 4. Discussion

The objective of this study was to analyze the changes produced in the lifestyle of pregnant women instituted as a result of the COVID-19 pandemic compared to their situation before the confinement. This study analyzed data obtained through an internet-based survey, and to the best of our knowledge, is the first to analyze changes in lifestyle caused by the confinement in this specific population group. The survey was disseminated among pregnant women in Valencia (Spain) after four weeks of government-imposed confinement, from 18 May to 31 May 2020, when Spain’s strictest restrictions on individual mobility had started to be eased. 

There has been much speculation about the possible repercussions of the confinement, especially on the population’s eating habits as a whole. These concerns were promoted by possible limitations in the availability of food and difficulties related to the daily purchase of fresh products, among other factors. In fact, some studies conducted during the COVID-19 outbreak have already shown that there were changes in diet quality among some population groups. For example, Pietrobelli et al. [52] observed an increase in the consumption of processed foods, such as potato chips and sugary drinks in obese Italian children. Other researchers reported an increase in the frequency and consumption of snacks and carbohydrate-rich foods in diabetic Indian patients [53]. However, despite the possibility that pregnant women might also change the type of diet they ate, we found that confinement had not affected their level of adherence to the MD. It is worth noting that high adherence to the MD is associated with beneficial effects during pregnancy in terms of preventing gestational diabetes, excess weight or obesity, and excess gestational weight [54,55]. Even though the results of this study showed that pregnant women did not change their eating habits due to confinement resulting from the COVID-19 pandemic, their adherence to the MD was lower than desirable. 

If we analyze the distribution of the participants according to their specific MD adhesion classification, more than half of these pregnant women (57.8%) showed a moderate adherence, around a third (35.6%) had high adherence, and only a small percentage (6.6%) reported low adherence. These results showed that the diet of approximately two-thirds of the participants was poorer than the national recommendations (SENC), in which high adherence to the MD is considered optimal. These results concur with those obtained in previous studies carried out in Spain with pregnant women, in which adherence to the MD was lower than recommended [56,57,58]. Specifically, a longitudinal study in which 793 pregnant women participated showed that, on average, moderate adherence to the MD was maintained throughout pregnancy [56]. Moreover, Cuervo et al. [57] carried out a cross-sectional study that included 13,845 women and found that their consumption of foods rich in protein, dairy products, vegetables, and cereals was lower than recommended. Another cross-sectional study observed that around 50% of pregnant women did not meet the recommendations for the consumption of cereals, legumes, milk, and dairy products [58]. 

Regarding exercise, the results of this study showed that there was a significant decrease in engagement in all three types of physical activity (vigorous, moderate, and walking) by these participants during the confinement, compared to their lifestyle habits during pregnancy from before the confinement. Indeed, when these women were asked if they thought the confinement had decreased their levels of physical activity, 87.7% answered yes, compared to 12.3% who indicated that they had not changed their physical activity habits. In contrast, only a third of those surveyed indicated that they had continued with their physical exercise routine, compared to a large percentage that had encountered some obstacles to exercising during confinement; among the most frequent obstacles reported were a lack of space (21.2%), fatigue due to the pregnancy (21.2%), and not considering exercise as a priority (12.2%). A small percentage of the participants did not engage in physical exercise out of fear to harm the fetus, a medical contraindication (risk of preterm birth), or another unspecified cause. This decrease in general physical activity was also associated with an increase in the number of hours spent sitting, which doubled.

The WHO recommends that adults aged 18–64 years engage in at least 150 min of moderate exercise or at least 75 min of vigorous exercise, or a combination of the two, spread throughout the week [49]. The guidelines also highlight that pregnant women may need to take additional precautions when exercising. Thus, the specific clinical guidelines for pregnant women rule out sports that imply the risk of falling, trauma, or collisions, and limit vigorous activity [59]. For example, the Spanish guidelines state that pregnant women should limit vigorous physical activity to no more than 15 min per day [60]. Despite the differences between the clinical guidelines from different countries regarding the frequency and optimal amount of exercise recommended during pregnancy, the general recommendations establish a minimum of 150 min of moderate exercise, preferably distributed throughout the week [59].

Moreover, the clinical guidelines in Spain also establish specific medical contraindications to engagement in exercise in pregnant women [59]. Among others, these include persistent bleeding, cardiovascular disease, cervical insufficiency, multiple pregnancies, preeclampsia or pregnancy-induced hypertension, and ruptured membranes. In this current study, 5.5% of the participants indicated having some medical contraindication for exercise, which would justify their reduced levels of reported physical activity. 

Studies carried out in several countries including Canada [61], Denmark [62], Norway [63], Japan [59], and Spain [42,60] have shown that pregnant women generally engage in less than the recommended minimum levels of exercise, even under normal conditions. In this current study, pregnant women spent an average of 68 min per week engaging in moderate physical activity during confinement, compared to 94 min before the COVID-19 pandemic, thus representing a reduction of nearly 30% compared to their previous levels. On average, these women had reduced their engagement in vigorous activity from twice a week to only once, completing an average of only 20 min per week. These results are below the recommended minimum levels of exercise. The frequency and time they spent walking also significantly decreased. Specifically, they went from walking an average of 98 min per week to only 38 min per week, representing an almost 60% decrease. This data is especially important if we consider that many women choose to walk at a fast pace as their primary physical activity during pregnancy [43,44]. Although some countries, such as Belgium, established looser confinement rules during the height of the COVID-19 pandemic, which allowed citizens to engage in physical exercise in the open air [64], the measures imposed in Spain were much more restrictive. The Spanish government banned all ‘non-essential’ outdoor activities for around a month, and citizens were encouraged to train indoors during this time. This fact justifies the decrease in the general levels of physical activity and an increase in the number of hours spent sitting that we observed in this study during the period of confinement.

The pregnant women included in this work also showed a significant decrease in HRQoL during the confinement, both in the five dimensions of the EQ-5D (mobility, self-care, usual activities, pain or discomfort, and anxiety or depression) and in the perception of health evaluated by the EQ-VAS. However, it is essential to consider that the decrease in HRQoL could be related to the evolution of the pregnancy itself. In this sense, other previous studies have also observed that HRQoL decreased as the pregnancy progressed and that this metric improved postpartum [65,66]. However, the decrease in HRQoL reported in this study may have been aggravated by the stress and anxiety produced by the situation of confinement itself [24,67,68,69]. In agreement with our results, some authors have also observed an increase in anxiety and depression levels during confinement in pregnant women [67,68] and in the immediate postpartum period [69]. Wu et al. analyzed the anxiety and depression of 4124 Chinese women during the outbreak of coronavirus. Their results showed an increase of anxiety and depression symptoms during confinement, especially in primiparous women, and a higher percentage of women with thoughts of self-harm [67]. Other studies carried out on Italian pregnant women have also observed a significant negative impact on the mental health of pregnant women as a consequence of confinement by COVID-19 [68]. 

On the one hand, Rashidi, Fakari and Simbar underlined anxiety and depression as worrying factors in this population group [45], and noted that fear of possible contagion was among the main concerns reported by pregnant women during this time. In this study, 22.5% of the women we surveyed reported having canceled an appointment for fear of contagion with SARS-CoV-2. On the other hand, some authors have observed that attending childbirth preparation classes helped decrease the levels of anxiety felt by expectant mothers [70]. In this regard, 90% of the respondents considered perinatal preparation important. However, during the confinement, 50% of the participants could not participate in these classes because they had been canceled due to the pandemic, while another 25% indicated that they had been able to do these classes online. In the anxiety/depression dimension of the EQ-5D, 41.1% of the participants showed moderate to extreme problems during confinement than a relatively small percentage (7.8%) who had experienced these symptoms during pregnancy before the confinement. 

The results in this study regarding lifestyle, as well as the perceived obstacles to engaging in exercise and preparing for childbirth during the COVID-19 confinement in Spain, suggest that specific online programs should be developed in order to promote healthy lifestyle habits and improve the HRQoL of pregnant women who may find themselves in similar circumstances in the future. In other population groups, such as obese patients, it has already been shown that the efficacy of these types of internet-based interventions is similar to that of traditional interventions [71,72,73]. The fact that online interventions can be self-administered and reach a broad audience because they are easy to access and have an excellent cost-benefit ratio [74] makes them an attractive tool for development and use in future situations. In this regard, future randomized controlled trial studies should evaluate the impact of online interventions based on healthy lifestyles (physical activity and nutrition) in face of possible new situations of confinement or restriction of individual mobility. 

We must mention that this study has some limitations, including social desirability bias and recall bias. Some authors suggest that investigating lifestyles through questionnaires can condition the responses, thus producing a tendency among participants to exaggerate their healthy habits and minimize their harmful habits [75]. Recall bias (the fact that responses depend on the participants’ ability to remember past events) has also been observed in retrospective cohort studies that used questionnaires to measure dietary intake [76,77] and often causes participants to underestimate their food consumption and risk of disease [76]. Besides, our participants were recruited from a public hospital (rather than a private one), which may have influenced our results due to sociodemographic status. On the other hand, it should be bore in mind that the MEDAS questionnaire collects information on the quality of the diet but does not allow us to know the levels of caloric intake of the participants. Finally, the sample size included in this work—based on assumptions—was not remarkably high; however, we have also to consider that the survey was disseminated only for 15 days, before the relaxation of the restriction measures in Spain.

## 5. Conclusions

The results of this study provide new data on the lifestyle changes made by pregnant women as a consequence of the confinement imposed because of COVID-19. Confinement decreased both the level of vigorous and moderate physical activity that these pregnant women had engaged in, as well as the time they spent walking, and doubled the number of hours they spent sitting. The main obstacles to engagement in physical activity reported by these women were a lack of space, fatigue due to pregnancy, and not considering exercise a priority. The confinement also negatively affected their HRQoL. Furthermore, 54.2% of the participants were unable to continue or start their preparation for delivery, while 24.4% of those surveyed participated in online perinatal preparation classes. Finally, this population group did not change their adherence to the MD due to social distancing and isolation resulting from the confinement.

## Figures and Tables

**Table 1:** Participants’ general characteristics.

Age (years)	33.1 ± 4.6
Height (cm)	164.2 ± 5.7
Weight pre-confinement (kg)	67.7 ± 17.5
Weight during confinement (kg)	72.96 ± 17.0
BMI (kg/m^2^) pre-confinement	25.0 ± 6.3
BMI (kg/m^2^) during confinement	27.0 ± 5.7
Week of pregnancy (number)	29.9 ± 8.3
Parity	
Primiparous	60 (66.6)
Multiparous	30 (33.3)

Values are expressed as mean ± standard deviation and as number and percentage [*n* (%)] for parity.

**Table 2:** Self-perception and perceived obstacles to exercise, preparation for delivery, and medical monitoring of their pregnancy during the confinement.

Self-Perception of Decreased Exercise During Confinement	79 (87.7)
**Reported obstacles to exercise**	
None	29 (32.2)
Lack of space	19 (21.2)
Fatigue from pregnancy	19 (21.2)
Not a priority	11 (12.2)
Fear of harming the fetus	4 (4.4)
Contraindication for risk of preterm birth	5 (5.5)
Others (no specified)	3 (3.3)
Extent to which preparation for delivery was considered important	81 (90)
**Reported obstacles to preparation for delivery**	
Cancellation of classes	47 (52.2)
Performing online classes	22 (24.4)
Too early to start classes	20 (22.2)
**Reported obstacles to attending medical appointments**	
Cancellation of medical appointments for fear of contagion	25 (22.5)

Values are expressed as number and percentage in parenthesis [*n* (%)].

**Table 3:** Results of the Wilcoxon tests for continuous variables.

	Pre-COVID-19	During-COVID-19	*p*	Z
Vigorous activity (days per week)	2 (3)	0 (2)	0.001	−3.345
Vigorous activity (min per day)	60 (70)	0 (30)	<0.001	−4.405
Moderate activity (days per week)	3 (3)	3 (3.5)	0.009	−2.581
Moderate activity (min per day)	60 (80)	60 (60)	<0.001	−3.464
Walking activity (days per week)	7 (2)	3 (6)	<0.001	−5.337
Walking activity (min per day)	90 (60)	30 (60)	<0.001	−5.507
Number of hours spent sitting per day	4 (4)	8 (5)	<0.001	−6.436
EQ VAS score	90 (1)	70 (2)	<0.001	−5.987

Values are expressed as medians and interquartile ranges in parenthesis.

**Table 4:** Number and proportion of reporting levels within EQ-5D dimensions: pre- and during COVID-19 confinement.

	Mobility		Self-Care		Usual Activities		Pain/Discomfort		Anxiety/Depression	
	*Pre- COVID-19*	*During-COVID-19*	*Pre- COVID-19*	*During-COVID-19*	*Pre- COVID-19*	*During-COVID-19*	*Pre- COVID-19*	*During-COVID-19*	*Pre- COVID-19*	*During-COVID-19*
LEVEL 1	90 (100)	70 (77.8)	89 (98.9)	81 (90)	84 (93.3)	50 (55.6)	75 (83.3)	36 (40)	83 (92.2)	53 (58.9)
LEVEL 2	0 (0)	19 (21.1)	0 (0)	9 (10)	6 (6.7)	36 (40)	14 (15.6)	49 (54.4)	6 (6.7)	36 (40)
LEVEL 3	0 (0)	1 (1.1)	1 (1.1)	0 (0)	0 (0)	4 (4.4)	1 (1.1)	5 (5.6)	1 (1.1)	1 (1.1)
TOTAL	90 (100)	90 (100)	90 (100)	90 (100)	90 (100)	90 (100)	90 (100)	90 (100)	90 (100)	90 (100)

Values are expressed as number and percentage in parenthesis [*n* (%)].

**Table 5:** Adherence to the MD and positive answers to the MEDAS questionnaire.

Adherence to the MD	Pre-COVID-19	During-COVID-19
Low	3 (3.3)	6 (6.7)
Medium	53 (58.8)	52 (57.8)
High	34 (37.8)	32 (35.6)
Olive oil, main dressing	87 (96.7)	86 (95.6)
Olive oil, ≥ 4 tsp./day	61 (67.8)	62 (68.9)
Vegetables, ≥ 2 s/day	59 (65.6)	62 (68.9)
Fruits, ≥ 3 s/day	36 (40)	45 (50)
Read meat, < 1 s/day	79 (87.8)	76 (84.4)
Butter, < 1 s/day	89 (98.9)	81 (90)
Sweet beverage, < 1 s/day	85 (94.4)	84 (93.3)
Wine, 7 s/week	0 (0)	0 (0)
Legumes, ≥ 3 s/week	22 (24.4)	24 (26.7)
Fish and seafood, ≥ 3 s/week	26 (28.9)	20 (22.3)
Sweets, < 3 s/week	60 (66.7)	52 (57.8)
Nuts, ≥ 3/week	57 (63.3)	63 (70)
White or red meat	73 (81.1)	73 (81.1)
Sofrito	56 (62.2)	60 (66.7)

Data are expressed as number and a percentage in parenthesis (*n* [%]). MD, Mediterranean diet; s, serving; tsp., tablespoon.
